# Implementation research in Primary Care (Part 2): How to conduct implementation research?

**DOI:** 10.51866/cm.1069

**Published:** 2026-01-31

**Authors:** Wen Ting Tong, Chor Yau Ooi, Anne Sales, Chirk Jenn Ng

**Affiliations:** 1 Department of Primary Care Medicine, Faculty of Medicine, Universiti Malaya, Kuala Lumpur, Malaysia.; 2 Department of Family Medicine, Faculty of Medicine and Health Sciences, Universiti Malaysia Sarawak, Kota Samarahan, Sarawak, Malaysia.; 3 Sinclair School of Nursing and Department of Family and Community Medicine, MU School of Medicine, University of Missouri, Missouri, USA.; 4 Centre for Population Health Research and Implementation, SingHealth Regional Health System, Singapore.

**Keywords:** Implementation science, Implementation research, Dissemination and implementation, Primary care, Family medicine

## Abstract

This paper is the second paper on implementation research in primary care. This paper outlines key processes for conducting implementation research, emphasising the importance of selecting relevant theories, models, and frameworks to guide each stage. Three core steps common for implementation research were described: identifying determinants that influence implementation, mapping appropriate strategies to address these determinants, and evaluating implementation outcomes. Determinant frameworks such as the Consolidated Framework for Implementation Research and the Theoretical Domains Framework help researchers understand contextual barriers and facilitators, while resources like the ERIC taxonomy and Behaviour Change Technique Taxonomy support strategy selection. Evaluation frameworks, including Proctor’s implementation outcomes and RE-AIM, enable systematic assessment of implementation success. This paper also highlights the essential role of stakeholder engagement throughout the research process. Together, these components offer a structured, theory-informed approach to support effective, scalable, and sustainable implementation efforts in primary care.

## Introduction

Implementation research is a powerful tool to support the adoption, and scale-up of interventions and their integration into health systems. Implementation research helps to identify the context where effective implementation can take place and support the process of iterative refinement needed for successful adaptation. Nevertheless, conducting implementation research is often viewed as a complex endeavour as it involves multiple phases, a wide range of approaches, various stakeholders, and outcomes.^1^ Nevertheless, as the field progresses, there is now a clearer guidance on how implementation research can be systematically conducted to achieve effective outcomes.

In our previous article, we introduced the concept of implementation research, highlighted its importance in advancing the field of primary care, and described its characteristics in comparison with the more well-known quality improvement approach, which is more commonly adopted among healthcare providers in improving healthcare delivery. In this second part of the article “Implementation Research in Primary Care”, we provide step-by-step guidance on how to undertake implementation research focussing on the key processes in conducting implementation research.

## Frameworks for conducting implementation research

In implementation research, the use of frameworks is crucial as they can help in planning, developing relevant implementation strategies, clarifying key constructs in the various phases of implementation research, and help to understand why a certain implementation succeed or fail in real-world practice.^2^ Currently, there are more than 100 frameworks that have been developed to guide implementation research.^3-5^ To guide the selection of an appropriate and relevant framework for implementation research, Nilsen et al., (2015) divided implementation frameworks into five categories: 1) process models, 2) determinants frameworks, 3) classic theories, 4) implementation theories, and 5) evaluation frameworks.^2^ Among these five categories, the process model, determinant, and evaluation frameworks were more commonly used.

Process models are frameworks that describe and guide the process of translating research into practice. As the name implies, process models offer practical step-by-step processes in the planning and execution of implementation research. Process models prescribe the steps that should be followed in the process of translating research into practice, from discovery and production of research-based knowledge to implementation and use of research in various settings.^2^

Determinant frameworks are frameworks that can help to identify barriers and facilitators, or factors influencing implementation outcomes. Identifying what influences implementation success is important for developing implementation strategies to address the relevant determinant for effective implementation.

Evaluation framework is a category of framework that provides a structure for evaluating an implementation endeavour.^2^ Evaluation frameworks specify the constructs that can be evaluated when assessing implementation outcomes.

These typologies proposed by Nilsen et al (2015) is a functional typology, and depending on the objective of the implementation endeavour, researchers can select a framework based on what purpose they want to use the framework for.^2^

## Steps in implementation

A process model provides step-by-step processes for conducting an implementation research. Comparing various process models, such as the Knowledge to Action Framework,^6^ the EPIS model (Exploration, Preparation, Implementation, Sustainment),^7^ Implementation Change Model,^8^ and implementation mapping,^9^ the three common steps are exploration of determinants (or barriers and facilitators) influencing implementation, mapping strategies to barriers, and evaluation. These steps have also been reported in a systematic review for design of implementation intervention to change healthcare providers’ behaviours.^10^

It is important to note that conducting implementation research is not always a linear process; a researcher can begin at different stages of implementation depending on the research question, and, therefore, different frameworks for different stages should be adopted. For example, a determinant framework such as the Theoretical Domains Framework (TDF) can be used to explore factors influencing implementation step before or after implementation, while for the evaluation step, the RE-AIM framework can be utilised (Table 1).

**Table 1:** Steps in implementation and their relevant framework, objective, and examples

Implementation stage	Relevant framework	Objective	Examples of implementation frameworks
Exploring factors influencing implementation	• Determinant framework	• To identify potential determinants (barriers and facilitators) before and/or after implementation.	Consolidated Framework for Implementation Research (CFIR) Theoretical Domains Framework (TDF) Tailored Implementation in Chronic Diseases (TICD)
Mapping strategies to barriers	• Repositories of implementation strategies	• To identify the strategies in addressing barriers or enhancing facilitators to implementation.	The Expert Recommendations for Implementing Change (ERIC) taxonomy Michie Behaviour Change Technique Taxonomy version 1 (BCCTv1) The CFIR-ERIC Implementation Strategy Matching Tool
Evaluation	• Evaluation framework	• To evaluate the implementation outcomes i.e. whether the healthcare intervention has been implemented successfully.	RE-AIM PRECEDE-PROCEED model Proctor’s implementation outcomes

In the following sections, detailed information is provided on how to select an appropriate framework to guide implementation, how to identify factors (barriers and facilitators) influencing implementation: what are the approaches and resources that can be used to develop an implementation intervention (map strategies to barriers), how to perform evaluation on an implementation study, and the ways to involve stakeholders in the implementation endeavour for effective outcome.

## Selecting an appropriate framework

Theoretical underpinning of implementation is important to achieve a more successful implementation as frameworks can help to understand and explain influences on implementation outcomes. Frameworks also help to provide a comprehensive and a more structured approach to implementation, ensuring accumulation of evidence by providing a consistent, replicable, and theory-informed structure, provide the common language and facilitate effective communication across various implementation key stakeholders.^11-13^

It is important that implementation researchers are aware of the various specific frameworks that are available (Table 1) to know which one is more appropriate to achieve the intended outcomes of the implementation research. The D&I Models Webtool lists various frameworks for implementation research, and they are categorised into functions (dissemination and/or implementation), target setting (individual community or system level), and relevance to the content area of study and /or context.^4^ Now, there is available literature that can help with the selection of framework.^2,4,14^ The Theory Comparison and Selection Tool (T-CaST) is another tool that can be used to select the most appropriate framework by comparing and scoring the potential framework to be used based on four constructs: applicability, usability, testability, and acceptability.^14^

## Exploring determinants (barriers and facilitators) influencing implementation

Understanding contextual factors influencing implementation is crucial to ensure that the strategies for implementation are optimised thereby increasing the likelihood of successful implementation.^15^ Among the common determinant frameworks are the Consolidated Framework for Implementation Research, the Theoretical Domains Framework, and Tailored Implementation for Chronic Diseases. The Consolidated Framework for Implementation Research (CFIR) is one of the most used determinant frameworks. It originated from the field of health services,16 and covers five domains: 1) intervention, 2) individuals, 3) inner setting, 4) outer setting, and 5) implementation processes. Under these five constructs there are 39 subconstructs.^16^

The TDF is an overarching framework of 14 theoretical domains synthesised from behaviour change constructs found in 33 behaviour change theories.^17^ It was developed to identify and describe factors that influence healthcare providers’ behaviours in relation to implementation of health innovation.^17^ Given the origin of how the framework was developed, the TDF focusses on individual behaviour influencing implementation and therefore includes constructs such as individuals’ knowledge, skills, beliefs about capabilities, and optimism that can influence implementation.^17^ TDF is more suitable for implementation research that emphasizes individual behaviour change as compared to CFIR, which focuses more on the organizational level.

The TICD checklist was developed based on a systematic review of frameworks of determinants of practice and consensus among implementation researchers.^18^ The development of this checklist was part of the TICD project, which aimed to assess the effectiveness of tailored programmes to improve healthcare for patients with chronic diseases and at the same time advancing knowledge on the concepts and methods of tailoring interventions to the identified determinants. The checklist comprises 57 potential determinants of practice grouped in seven domains: guideline factors, individual health professional factors, patient factors, professional interactions, incentives and resources, capacity for organisational change, and social, political, and legal factors.^18^

## Mapping strategies to barriers

Implementation strategies are ‘methods or techniques used to enhance the adoption, implementation, and sustainability of a clinical programme, practice or intervention’.^19^ According to Curran et al., (2020), implementation strategies are the “stuff we do” to try to help people and organizations to do “the thing”.^20^ For example, the strategy ‘audit and feedback’, through action enablement, training, and environmental restructuring, has been shown to improve adherence to guideline for improving acute stroke treatment practices.^21^ In tailored implementation, strategies are selected and mapped to address the barriers and facilitators to implementation within specific contexts. Often in implementation research, discrete implementation strategies are bundled to form an implementation intervention.

There are various resources for selecting implementation strategies, and a commonly used one is the Expert Recommendations for Implementing Change (ERIC) taxonomy, which comprises 73 strategies categorised into nine groups: 1) use evaluative and iterative strategies, 2) provide interactive assistance, 3) adapt and tailor to context, 4) develop stakeholder interrelationships, 5) train and educate stakeholders, 6) support clinicians and healthcare professionals, 7) engage patients/service users, 8) utilise financial strategies, and 9) change infrastructure.^22,23^ Another implementation strategy resource is the Michie Behaviour Change Technique Taxonomy version 1 (BCTTvl), which is comprised of 93 BCTs.^24^ However, it is different from the ERIC taxonomy in that the strategies are comprised of behaviour change techniques (BCTs) and are used more for development of implementation intervention at an individual level. BCTs are defined as *“observable, replaceable, irreducible component of an intervention designed to alter or redirect causal processes that regulate behavior”?'^1^* BCTs are behavior change strategies and are described as “active ingredients” that can cause behavior change.^24^ There is now an online resource known as the Theory & Techniques Tool that provides information on BCT-MoA links that can guide in the selection of BCT as part of intervention development.^25^ Currently, the understanding of which strategy can address a specific implementation barrier, and how strategies exert their effects remain understudied. Hence, more research is needed in guiding the selection of implementation strategy when developing an implementation intervention.

Other important considerations when mapping strategies to barriers include the use of theoretical or conceptual reasoning, engagement of stakeholders (e.g., healthcare providers, patients, public), and selection based on criteria such as evidence on effectiveness, feasibility, or importance. An example of a structured evaluation method is APEASE, a set of criteria used in designing behavioural change interventions to assess their suitability. The acronym stands for Acceptability, Practicability, Effectiveness, Affordability, Side-effects/safety, and Equity. These factors ensure that an intervention is not only impactful but also take into considerations resource constraints, ethical and social implications. In clinical practice, APEASE is utilized to evaluate intervention strategies aimed at promoting evidence-based practices among healthcare providers. For instance, in efforts to enhance hand hygiene in hospitals, APEASE can help determine whether providing alcohol gel at each bedside (which is practical, acceptable, and effective) is a better option than coercive measures, which may be less feasible and well-received by the healthcare providers. By systematically applying the APEASE criteria, interventions can be designed to maximize effectiveness while remaining practical and ethically sound in real-world healthcare settings.^13^

The implementation research logic model (IRLM) is another useful tool that can be used to help develop implementation intervention.^26^ Through the exercise of mapping strategies to address determinants that can lead to expected outcomes, the IRLM enables understanding on how and why an implementation is successful or fail by identifying the causal pathways between determinants, strategies, and outcomes.^26^ The IRLM is a semi-structured tool that allows implementation researcher to link their study determinants (barriers/facilitators), implementation strategies, mechanisms of action, implementation outcomes, and clinical outcomes.

## Evaluation

Proctor et al., (2011) highlighted that there is a need for distinction between implementation, service system and client outcomes.^27^ Service outcomes measure healthcare delivery aspect such as care efficiency, safety, effectiveness, equity, patient centeredness, and timeliness while client outcomes measure patient experience and clinical outcomes including satisfaction, function, health status, and symptoms. These measures are typically found in the clinical efficacy/effectiveness studies. In IS, outcomes of interest must include implementation outcomes, defined as *“the effects of deliberate and purposive actions to implement new treatments, practices, and services”?^1^*

Implementation outcomes are proximal indicators of implementation processes and should serve as intermediate outcomes to achieve effective service and/or client outcomes. When an innovation is implemented well and according to plan, it is more likely to lead to its desired end outcomes. As a result, implementation outcomes are indicators of implementation success.^27^ Implementation outcomes help to assess how the implementation processes and strategies influence the service and/or client outcomes. They guide selection of strategies by indicating what needs to be improved for effective implementation such as acceptability, feasibility, fidelity, or sustainability to make the innovation successful in practice. Implementation outcomes include acceptability, adoption, appropriateness, costs, feasibility, fidelity, penetration, and sustainability.

Another common framework for implementation evaluation is the RE-AIM framework. Originated from the field of public health, it was intended to improve the reporting of findings of implementing public health innovations by getting researchers to be more transparent and consider internal and external validity when conducting efficacy and translational research. RE-AIM expands the usual traditional clinical or treatment outcome measurements (i.e., efficacy/effectiveness) to implementation outcomes that are critical for broader impact. RE-AIM is an acronym, which stands for Reach, Effectiveness, Adoption, Implementation, and Maintenance.^28^ RE-AIM can help to measure whether the innovation reached to the intended audience (Reach), if the innovation was effective in meeting its outcomes (Effectiveness), user’s adoption of the innovation (Adoption), whether the users implemented the innovation as intended in the implementation protocol (Implementation), and if the implementation was sustainable (Maintenance). The framework can also help to identify which aspect of the implementation needs to be improved. It should be noted that some domains across different evaluation frameworks are similar to each other. For example, the domain ‘Implementation’ in RE-AIM is similar to Proctor’s evaluation outcome ‘Fidelity’, in which both aim to assess adherence to the implementation protocol.

## Other considerations

### Involvement of stakeholders

Involvement of stakeholders is a crucial component for implementation research as this ensures that the implementation research questions and priorities are relevant and aligned with diverse stakeholders’ needs and preferences, thereby increasing the likelihood that the intervention can be feasibly implemented, adopted, and sustained. Involvement of stakeholders is also an indicator of high-quality research where stakeholders are involved to ensure outcomes are important and relevant to them, not just the researchers.^29-31^

Implementation research usually involves a range of stakeholders with different backgrounds, knowledge and skills. Concannon et al., (2012) developed the 7Ps Framework to identify stakeholders,^32^ and the key groups are listed in Table 3.

**Table 3:** The 7Ps Framework to Identify Stakeholders^32^

Category	Description
Patients and the public	Patient, caregivers, families, consumers of health care
Providers	Those that provide care to patients and populations: Providers of care and support services (e.g., doctors, nurses, physicians, mental health counsellors, pharmacists, and other) Healthcare organizations (e.g., hospitals, clinics, community health centres, community-based organizations,)
Purchasers	Those responsible for underwriting the costs of health care (e.g.: employers, the self-insured, government and other entities
Payers	Those responsible for reimbursement for interventions and episodes of care (e.g.: insurers, individuals with deductibles)
Policy makers	Those responsible for making policies
Product makers	Drug and device manufacturers
Principal investigators	Researchers and their funders

Stakeholders should be treated as key implementation partners and be involved right from the start, and there is a need to consider who are the right stakeholders for the implementation work. This can be done using the stakeholder matrix and group the stakeholder based on their power (high and low) and interest (high and low).^33^ It is important to note that stakeholders may change as individuals within stakeholder organisations frequently move or change roles, and the goals of the implementation process may change over time.

Stakeholders can be involved in various ways in implementation research including identifying and formulating implementation research aims, guiding design and delivery of implementation strategies and evaluation plans, and partnering in dissemination, scale-up and spread of implementation project output.^32^ It is important that implementation researchers are clear on why they are involving the stakeholders, how they plan to incorporate their expertise, and be aware of the stakeholders expectations.^34^

Mechanisms for engaging stakeholders may include a modified Delphi or similar process, concept mapping, asynchronous web-based input, focus groups, surveys, but the choice of mechanism must be aligned as best possible with stakeholder preferences.^32^

### The inside-out change mindset

Implementing interventions in primary care practice has always been an outside-in approach where practices focused on achieving healthcare quality metrics, and meeting policy requirements. Such approach can lead to limited success and may cause more clinic disruptions and burnout. Hence, there is a need for inherent motivation to drive practice change among implementation researchers and work together with other stakeholders including healthcare authority to drive the transformation. Implementation research provides an approach that allow both “bottom-up” innovation, and “top-down” reforms. Where possible, implementation researcher should explore their own practice gaps, and co-create solutions with stakeholders to enable a more sustainable healthcare system to be built.^35^

Box 1 **Key points**

- There are more than 100 frameworks that have been developed to guide implementation research.
- Implementation frameworks can be divide into five categories: 1) process models, 2) determinant frameworks, 3) classic theories, 4) implementation theories, and 5) evaluation frameworks.
- It is important that implementation researchers select a framework that can be used optimally to ensure that the constructs are relevant for the implementation context, and the outcomes intended to achieve.
- Implementation research begins with selecting the appropriate process model that can provide practical guidance on the steps that need to be taken for their implementation work.
- Determinant frameworks are useful for understanding contextual factors that influence implementation.
- Implementation strategies are selected and mapped to address the barriers and facilitators to implementation. Discrete implementation strategies are bundled up to form an implementation intervention.
- Implementation evaluation is important to determine how the implementation processes and strategies influence the observed outcomes.
- Involvement of stakeholders ensures that the implementation research questions and priorities are relevant and aligned with the needs and preferences of target population and setting, thereby increasing the likelihood of sustained adoption of the intervention.

Box 2 **Useful resources**

- Bauer MS, Damschroder L, Hagedorn H, Smith J, Kilbourne AM. An introduction to implementation science for the non-specialist. BMC Psychol. 2015 Sep 16;3(1):32. doi: 10.1186/s40359-015-0089-9.
- Bauer MS, Kirchner J. Implementation science: What is it and why should I care? Psychiatry Res. 2020 Jan;283:112376. doi: 10.1016/j.psychres.2019.04.025.
- Nilsen, P. Making sense of implementation theories, models and frameworks. Implementation Sci 10, 53 (2015). https://doi.org/10.1186/s13012-015-0242-0
- The Implementation Science Exchange: https://impsci.tracs.unc.edu/
- The D&I Models Webtool is an interactive, online resource designed to help researchers and practitioners navigate D&I theories, models, and frameworks (TMFs) through planning, selecting, combining, adapting, using, and linking to measures.: https://dissemination-implementation.org/
- RE-AIM: https://re-aim.org
- CFIR: https://cfirguide.org
- Orientation to the Science of Dissemination and Implementation: https://cancercontrol.cancer.gov/is/training-education/orientation-to-the-science-of-dissemination-and-implementation

## Conclusions

Implementation research provides essential tools to bridge the gap between evidence and practice in primary care. By systematically selecting and applying appropriate theories, models, and frameworks, researchers can better understand contextual determinants, design tailored strategies and evaluate implementation outcomes. A structured process encompassing exploration of barriers and facilitators, mapping strategies to address them, and conducting rigorous evaluation enhances the likelihood of sustainable adoption and scale-up of healthcare innovations. Central to this process is the meaningful engagement of stakeholders, whose involvement ensures that implementation efforts are relevant, feasible, and responsive to real-world needs. As implementation research continues to evolve, its application in primary care offers a pathway to improving healthcare delivery, advancing equity, and strengthening health systems.

## References

[1] Tarrant C, O'Donnell B, Martin G (2016). A complex endeavour: an ethnographic study of the implementation of the Sepsis Six clinical care bundle.. Implement Sci.

[2] Nilsen P (2015). Making sense of implementation theories, models and frameworks.. Implementation Sci.

[3] Strifler L, Cardoso R, McGowan J (2018). Scoping review identifies significant number of knowledge translation theories, models, and frameworks with limited use.. J Clin Epidemiol.

[4] University of Colorado (2024). Explore D&I TMFs.. https://dissemination-implementation.org/tool/explore-di-models/.

[5] Wang Y, Wong EL, Nilsen P (2023). A scoping review of implementation science theories, models, and frameworks - an appraisal of purpose, characteristics, usability, applicability, and testability.. Implement Sci.

[6] Graham ID, Logan J, Harrison MB (2006). Lost in knowledge translation: time for a map?. J Contin Educ Health Prof.

[7] Moullin JC, Dickson KS, Stadnick NA (2019). Systematic review of the Exploration, Preparation, Implementation, Sustainment (EPIS) framework.. Implementation Sci.

[8] Grol R, Wensing M, Michel W, Richard G, Jeremy G (2020). Chapter 3 Effective Implementation of Change in Healthcare - A Systematic Approach.. Improving Patient Care: The Implementation of Change in Health Care, Third Edition.

[9] Fernandez ME, Ten Hoor GA, van Lieshout S (2019). Implementation Mapping: Using Intervention Mapping to Develop Implementation Strategies.. Front Public Health.

[10] Colquhoun HL, Squires JE, Kolehmainen N (2017). Methods for designing interventions to change healthcare professionals' behaviour: a systematic review.. Implementation Sci.

[11] Davidoff F, Dixon-Woods M, Leviton L (2015). Demystifying theory and its use in improvement.. BMJ Qual Saf.

[12] Davis R, Campbell R, Hildon Z (2015). Theories of behaviour and behaviour change across the social and behavioural sciences: a scoping review.. Health Psychol Rev.

[13] Michie SF, West R, Campbell R (2014). ABC of Behaviour Change Theories.

[14] Birken SA, Rohweder CL, Powell BJ (2018). T-CaST: an implementation theory comparison and selection tool.. Implementation Sci.

[15] Nilsen P, Bernhardsson S (2019). Context matters in implementation science: a scoping review of determinant frameworks that describe contextual determinants for implementation outcomes.. BMC Health Serv Res.

[16] Damschroder LJ, Aron DC, Keith RE (2009). Fostering implementation of health services research findings into practice: a consolidated framework for advancing implementation science.. Implementation Sci.

[17] Cane J, O'Connor D, Michie S (2012). Validation of the theoretical domains framework for use in behaviour change and implementation research.. Implementation Sci.

[18] Flottorp SA, Oxman AD, Krause J (2013). A checklist for identifying determinants of practice: A systematic review and synthesis of frameworks and taxonomies of factors that prevent or enable improvements in healthcare professional practice.. Implementation Sci.

[19] Proctor E, Powell B, McMillen C (2013). Implementation strategies: recommendations for specifying and reporting.. Implementation Sci.

[20] Curran GM (2020). Implementation science made too simple: a teaching tool.. Implement Sci Commun.

[21] Springer MV, Sales AE, Islam N (2021). A step toward understanding the mechanism of action of audit and feedback: a qualitative study of implementation strategies.. Implementation Sci.

[22] Powell BJ, Beidas RS, Lewis CC (2017). Methods to Improve the Selection and Tailoring of Implementation Strategies.. J Behav Health Serv Res.

[23] Waltz TJ, Powell BJ, Matthieu MM (2015). Use of concept mapping to characterize relationships among implementation strategies and assess their feasibility and importance: results from the Expert Recommendations for Implementing Change (ERIC) study.. Implementation Sci.

[24] Michie S, Richardson M, Johnston M (2013). The behavior change technique taxonomy (v1) of 93 hierarchically clustered techniques: building an international consensus for the reporting of behavior change interventions.. Ann Behav Med.

[25] Johnston M, Carey RN, Connell Bohlen LE (2020). Development of an online tool for linking behavior change techniques and mechanisms of action based on triangulation of findings from literature synthesis and expert consensus.. Transl Behav Med.

[26] Smith JD, Li DH, Rafferty MR (2020). The Implementation Research Logic Model: a method for planning, executing, reporting, and synthesizing implementation projects.. Implementation Sci.

[27] Proctor E, Silmere H, Raghavan R (2011). Outcomes for implementation research: conceptual distinctions, measurement challenges, and research agenda.. Adm Policy Ment Health.

[28] RE-AIM Workgroup (2020). RE-AIM.. http://www.re-aim.org/about/frequently-asked-questions/.

[29] Burton C, Rycroft-Malone J (2015). An Untapped Resource: Patient and Public Involvement in Implementation Comment on "Knowledge Mobilization in Healthcare Organizations: A View From the Resource-Based View of the Firm".. Int J Health Policy Manag.

[30] Callard F, Rose D, Wykes T (2012). Close to the bench as well as at the bedside: involving service users in all phases of translational research.. Health Expect.

[31] Ocloo J, Matthews R (2016). From tokenism to empowerment: progressing patient and public involvement in healthcare improvement.. BMJ Qual Saf.

[32] Concannon TW, Meissner P, Grunbaum JA (2012). A new taxonomy for stakeholder engagement in patient-centered outcomes research.. J Gen Intern Med.

[33] Mendelow A (1991). Stakeholder mapping.. Proceedings of the 2nd International Conference on Information Systems.

[34] Boaz A, Hanney S, Borst R (2018). How to engage stakeholders in research: design principles to support improvement.. Health Res Policy Syst.

[35] Miller WL, Rubinstein EB, Howard J (2019). Shifting Implementation Science Theory to Empower Primary Care Practices.. Ann Fam Med.

